# Prognostic 7-SLC-Gene Signature Identified via Weighted Gene Co-Expression Network Analysis for Patients with Hepatocellular Carcinoma

**DOI:** 10.1155/2023/4364654

**Published:** 2023-02-17

**Authors:** Lingfeng Xiong, Yongping Luo, Tianbai Yuan, Weipeng Lin, Bohui Lin, Chen Wu, Yuyou Duan, Yimeng Ou

**Affiliations:** ^1^Department of Hepatobiliary Surgery, The First Affiliated Hospital of Guangdong Pharmaceutical University, Guangzhou, China; ^2^Department of Thyroid and Breast Surgery, Ward 1, Weifang People's Hospital, Weifang, China; ^3^Laboratory of Stem Cells and Translational Medicine, Institutes for Life Sciences, School of Medicine, South China University of Technology, Guangzhou, China

## Abstract

**Background:**

Solute carrier (SLC) proteins play an important role in tumor metabolism. But SLC-associated genes' prognostic significance in hepatocellular carcinoma (HCC) remained elusive. We identified SLC-related factors and developed an SLC-related classifier to predict and improve HCC prognosis and treatment.

**Methods:**

From the TCGA database, corresponding clinical data and mRNA expression profiles of 371 HCC patients were acquired, and those of 231 tumor samples were derived from the ICGC database. Genes associated with clinical features were filtered using weighted gene correlation network analysis (WGCNA). Next, univariate LASSO Cox regression studies developed SLC risk profiles, with the ICGC cohort data being used in validation.

**Result:**

Univariate Cox regression analysis revealed that 31 SLC genes (*P* < 0.05) were related to HCC prognosis. 7 (SLC22A25, SLC2A2, SLC41A3, SLC44A1, SLC48A1, SLC4A2, and SLC9A3R1) of these genes were applied in developing a SLC gene prognosis model. Samples were classified into the low-andhigh-risk groups by the prognostic signature, with those in the high-risk group showing a significantly worse prognosis (*P* < 0.001 in the TCGA cohort and *P*=0.0068 in the ICGC cohort). ROC analysis validated the signature's prediction power. In addition, functional analyses showed enrichment of immune-related pathways and different immune status between the two risk groups.

**Conclusion:**

The 7-SLC-gene prognostic signature established in this study helped predict the prognosis, and was also correlated with the tumor immune status and infiltration of different immune cells in the tumor microenvironment. The current findings may provide important clinical indications for proposing a novel combination therapy consists of targeted anti-SLC therapy and immunotherapy for HCC patients.

## 1. Introduction

Liver cancer ranks as the second highest cause of tumor-resulted mortality [1]. Hepatocellular carcinoma (HCC) constitutes 90% of liver cancer cases. Despite significant advances in therapeutic approaches, the recurrence, progression, and metastasis rates of HCC remain high, leading to a poor HCC prognosis [2]. At present, the main treatment options available for HCC are systemic transplantation, drug therapy, transcatheter arterial chemoembolization and radiotherapy, ablative therapy, and surgical resection [3]. However, a great number of HCC patients are already at an advanced stage by the time of diagnosis. Due to the complex molecular mechanisms and cellular heterogeneity of HCC, traditional clinical indicators such as AFP, TNM staging, and vascular invasion have limited ability for predicting the prognosis of HCC. Therefore, for facilitating early detection, predicting the prognosis, and guiding individualized treatment, novel, and more accurate methods are required to understand more clearly HCC developmental mechanisms [4].

After G-protein-coupled receptors, the solute carrier (SLC) superfamily encodes the second largest membrane transporter protein and consists of 65 families and approximately 400 SLC transporter proteins that mainly maintain the stability of the intracellular environment through facilitating various soluble molecular substrates exchange across the lipid membrane [5]. Approximately 80% of small chemical molecules are functionally SLC proteins-dependent [6]. SLC proteins participate in various diseases, for instance, cardiovascular diseases, mental disorders, cancers, and some chronic diseases [7]. SLC proteins play different roles in tumor development via regulation of biological processes such as chemoresistance, angiogenesis, proliferation, EMT, metastasis, migration, and immunosuppression as well as the regulation of regulating different GFS, metalloproteinases (MMPs), TF, signaling cascades, and cytokines [8]. However, the role and significance of the SLC family in HCC was not completely clear. How genes are associated in different modules and clinical phenotypes could be systematically described by Weighed gene expression network analysis (WGCNA) [9]. Clinical data information of HCC patients with as well as their mRNA expression profiles were obtained publicly from databases. Subsequently, WGCNA was performed using data from the TCGA training cohort to screen module genes associated with tumor staging, and analysis of univariate and LASSO Cox data have both shown that SLC22A25, SLC2A2, SLC41A3, SLC44A1, SLC48A1, SLC4A2, and SLC9A3R1 were prognostic SLC markers, which were validated using data from the ICGC. To assess the underlying mechanisms of these genes, we then performed a functional enrichment analysis.

## 2. Materials and Methods

### 2.1. Acquisition of RNA-seq Data

The RNA-seq data (FPKM: fragments per kilobase of exon per million mapped fragments) and related clinical information of HCC patients originated from the TCGA (https://portal.gdc.cancer.gov/repository) and ICGC databases (https://dcc.icgc.org/projects/LIRI-JP). For this study, 231 HCC cases from the ICGC dataset and 371 HCC tissues, and 50 adjacent healthy tissues from the TCGA dataset were selected. Single-cell RNA sequencing (scRNA-seq) dataset (GSE149614) included 25,479 genes and 71,915 cells from the GEO database (https://www.ncbi.nlm.nih.gov/geo/query/acc.cgi?acc=GSE149614).

### 2.2. Co-Expression Network of SLC Family Genes

Previously, using the human gene database GeneCards (https://www.genecards.org/), SLC genes have been identified [10], and a co-expression network targeting the SLC family was constructed using the WGCNA R package (version 1.68) [9]. Initially, 397 genes of the SLC family in the TCGA-LIHC cohort were selected as input genes for network construction, and between a gene pair, the Pearson correlation similarity matrix was determined and increased to a soft threshold according to the scale-free topological network criteria. Following this, clustering of the adjacency matrix was carried out with topological overlap (1-TOM) plus dissimilarity (1-TOM). Furthermore, to identify gene modules on the dendrogram, a dynamic tree-cutting algorithm was introduced, with 30 being set as the minimum gene number in each module. In each module, module eigengene (ME) refers to the main component in the gene expression. A Pearson correlation was evaluated between MEs and clinical features (tumor stage, tumor grade, and AFP), and the most relevant modules were selected.

### 2.3. Developing and Validating a Gene Model with the SLC Family

Genes not included in the ICGC database were excluded from the modules. Our results from the univariate Cox regression study suggested a relation of SLC genes to a prognostic effect on overall survival; LASSO-Cox regression analysis was conducted subsequently for genes with *P* values of <0.05 via the glmnet R package [11] to avoid overfitting. The risk score of HCC patients was evaluated using the SLC risk score = Ʃ(*β*_*i*_^*∗*^Exp_*i*_), *β*_*i*_ is the LASSO coefficient of the gene, whereas Exp_*i*_ is the level of expression of a gene. Using the median risk score, training cohort patients were classified into groups of low-risk and high-risk. Subsequently, the difference in OS of the two groups was estimated based on Kaplan–Meier and ROC curves. Subsequently, the validation of the SLC risk model in the ICGC cohort was operated.

### 2.4. Genomics and Genome Studies Using KEGG and GO

To determine biological functions (which include cellular components [CCs], molecular functions [MFs], and biological processes [BPs]) and pathways (*P* values of <0.05 indicated significant enrichment), we analyzed Gene Ontology (GO) and Kyoto Encyclopedia of Genes and Genomes (KEGG) pathway enrichment analyses on genes identified using univariate Cox regression analysis in clusterProfiler R package (version 3.14.3) [12].

### 2.5. Analysis of scRNA-seq Data

ScRNA-seq analysis was carried out in the “Seurat” R package [13]. The study included at least 10,000 samples containing detected genes. As part of quality control (QC), the following criteria were introduced: (1) excluding genes detected in fewer than five cells; (2) excluding cells detected fewer than 200 genes overall. By normalizing the merged data first and then identifying variable features with the FindVariableFeature function, we collected the first 2000 highly variable genes (based on variance stabilization transformation). We also used the scale data function to scale all the genes, and principal meta-analysis with RunPCA function to reduce the dimensionality of the first 2000 highly variable genes screened. To find cell clusters, we chose DIM = 1 : 15 and used the functions “FindNeighbors” and “FindCluster” (resolution = 0.5). Next, DIM = 1 : 15 was selected and further downscaled using UMAP. Then, the FindAllMarkers function with logFC = 0.25 (difference ploidy) and minpct = 0.25 (expression ratio of minimum difference genes) was used to screen marker genes in 34 subgroups. In the final step, an adjusted *P* < 0.05 was used for screening marker genes. In addition to cluster classification, we identified and annotated the different cell clusters via “Celldex” and “Singler” packages in R. Then, the Monocle package [14] analyzed single-cell trajectory data to discover cell-state transitions and their relationship to the seven SLC genes.

### 2.6. Gene Enrichment Analysis

Using the enrichplot and clusterProfiler R packages, an analysis of gene set enrichment (GSEA) was conducted to identify KEGG pathway genes and enrich marker genes between risk groups. Molecular Signature Database (MSigDB) was used to obtain “c2.cp.kegg.v7.4.symbol” and “h.all.v7.4.symbol” gene sets [15].

### 2.7. Validation Using the Human Protein Atlas Database

Immunohistochemical (IHC) staining images of the markers genes in HCC tissues [16] from the Human Protein Atlas (HPA) database (https://www.proteinatlas.org/) was searched for, which allowed a direct observation of the localization of target proteins.

### 2.8. Estimation of Immune Cell Infiltration

Data regarding tumor immune cell infiltration were available on TISIDB (https://cis.hku.hk/TISIDB/download.php), and the ESTIMATE package in R was used to calculate immune scores, ESTIMATE scores, and tumor purity for each sample to quantify the tumor immune microenvironment. In addition, between the high- and low-risk groups of the TCGA and ICGC cohorts, differences in immune cell infiltration were compared.

### 2.9. The Correlation between the SLC Gene Signature and Immune Checkpoints

From the UCSC database (https://xenabrowser.net/), we downloaded the uniformly normalized pan-cancer data set TCGA TARGET GTEx (PANCAN, *N* = 19131, *G* = 60499), and the expression data of 60 genes of two types of immune checkpoint pathways (inhibitory, 24; stimulatory, 36 [17]) and the marker gene expression data of each sample were extracted from the dataset. The samples collected from primary solid tumors, primary tumors, and primary blood-derived cancer (bone marrow or peripheral blood) were screened. All healthy samples were refined, and their expression values were log2 (*x* + 0.001)-transformed. In addition, the Pearson correlation coefficients were calculated.

### 2.10. Somatic Alteration Data Collection and Analyses

Somatic alteration data of the TCGA training cohort were extracted from the Genomic Data Commons data portal (https://gdc.cancer.gov/about-data/publications/mc3-2017) [18], and the maftools R package [19] was used to identify and visualize low-risk and high-risk SLC mutations in the top 20 highest mutation frequencies.

### 2.11. Analysis of the Response to Chemotherapy Drugs

To determine the sensitivity of samples to various chemotherapeutic agents, R package pRRophetic predicted drugs with half-maximal inhibitory concentrations (IC50) in patients of HCC in different risk groups. Multiple studies have used this algorithm previously and have been widely published [20].

### 2.12. Statistical Analyses

R software (version R.4.1.0) performed all the statistical analyses in this study. A two-sided*P* value of <0.05 referred to a statistical significance. In paired comparisons, we employed the Wilcoxon test. To compare overall survival, log-rank tests, and Kaplan–Meier curves in this study were applied with the survival and survminer R packages.

## 3. Results

### 3.1. Identification of SLC Family Genes Associated with the Prognosis of HCC

**Figure 1:**
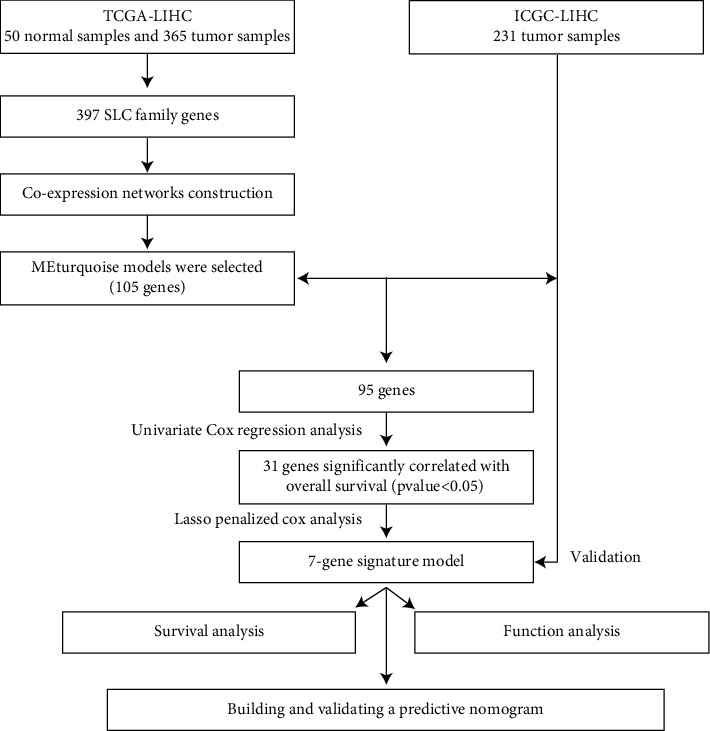
Workflow of this study.

Figure 1 shows the study flow chart. Ultimately, we included 231 patients with HCC from the ICGC (LIRI-JP) cohort and 365 patients with HCC from the TCGA-LIHC cohort. We identified 397 well-defined SLC genes, and their expression data were taken from the TCGA-LIHC dataset.

### 3.2. Co-Expression Network of SLC and Clinical Features

**Figure 2:**
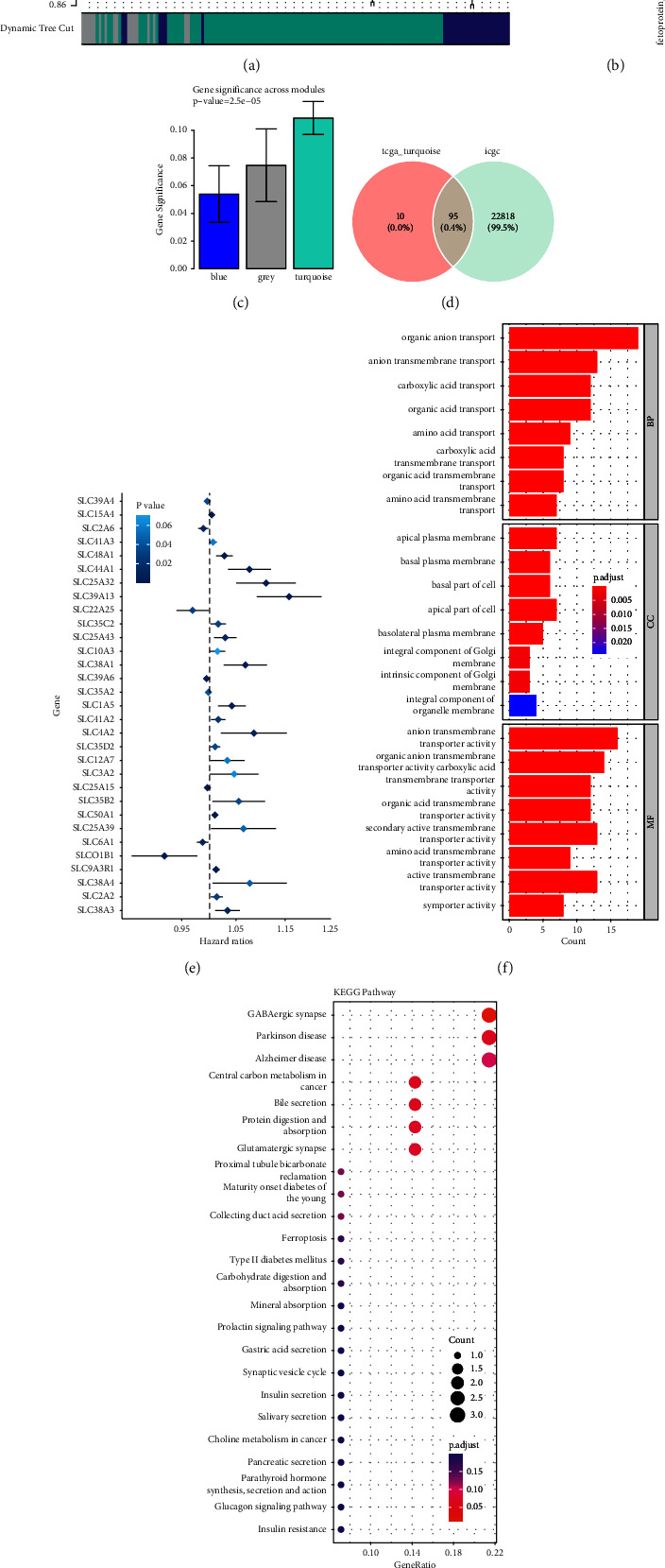
Identification of the candidate SLC-related genes in the TCGA cohort. (a) Clustering dendrograms showing genes with similar expression patterns were clustered into co-expression modules. Genes not assigned a module are indicated by gray modules. (b, c) The module-trait relationships reveal the relationship between each gene module eigengene and the clinical characteristics determined by the TCGA-LIHC. (d) The fraction of module genes not included in the ICGC cohort is shown in the venn diagram. (e) Gene expression along with OS is shown in forest plots between univariate Cox regression analysis. (f, g) Analyses of gene ontology (GO) that enriched the biological process (BP), cellular component (CC), and molecular function (MF), as well as a KEGG pathway analysis of the 31 genes.

WGCNA was performed using data from the TCGA-LIHC cohort. Three co-expression models were clustered in the hierarchical clustering tree (Figure 2(a)). According to the MEturquoise module, relatively strong positive correlations with tumor stage (Cor = 0.28, *P* = 4*e* − 7) and grade (Cor = 0.31, *P* = 3*e* − 8) were found (Figures 2(b) and 2(c)). A total of 105 genes were included in the MEturquoise module. As shown in Figure 2(d), 90.5% (95/105) genes in the MEturquoise module were co-expressed in the ICGC (LIRI-JP) dataset and were subsequently subjected to univariate Cox regression analysis. Thirty one prognosis-associated genes were identified (*P* value <0.05) (Figure 2(e)). Next, these 31 genes were subjected to GO and KEGG analyses. Organic anion transport was the main enriched BP term, whereas parietal plasma membrane and anion transmembrane transport protein activity were the main enriched CC and MF terms, respectively (Figure 2(f)). GABAergic synapses, central carbon metabolism in cancer, and bile secretion were significantly enriched KEGG pathways (Figure 2(g)).

### 3.3. Seven SLC Genes Were Verified in HCC by scRNA-seq Analysis

**Figure 3:**
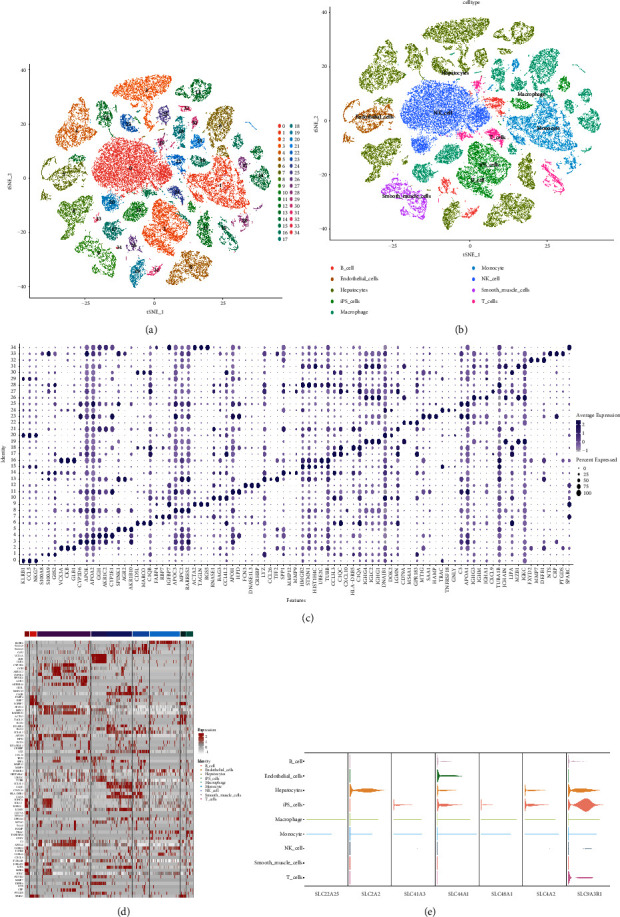
HCC cells are classified into 34 clusters and 9 types. (a) The tSNE plot shows 34 cell clusters. (b) The marker genes successfully identified nine cell types. (c) Based on the composition of marker genes, dot plots for 34 clusters were created by CellMarker. (d) Expression levels of specific markers in each cell type as shown on the heatmap. (e) Various cell types identified in HCC are represented by violin plots showing the expression of seven SLCs markers.

**Figure 4:**
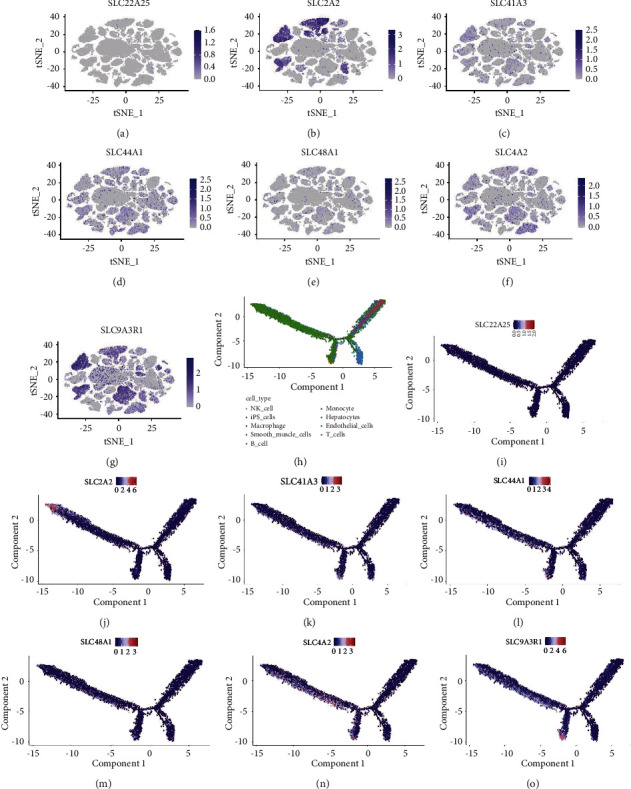
Seven-SLC genes distribution in the microenvironment of HCC. (a–g) Expression levels of 7 SLCs in the nine cell types. (h–o) Here is an unsupervised transcriptional trajectory of different cells from monocle colored according to their cell type (h) and their expression levels (i–o).

The dataset GSE149614 consists of 71,915 single cells that were then subjected to scRNA-seq analysis, and unsupervised classification was successful in classifying the cells into 34 clusters (Figure3(a)). These 34 cell clusters showed different expression patterns (Figure 3(c)). Our analysis of CellMarker markers determined the nine cell types using “celldex” and “SingleR” markers, namely, 1) hepatocytes; 2) B_cell; 3) endothelial_cell. 3) endothelial_cells; 4) iPS_cells; 5) macrophage; 6) monocyte; 7) NK_cell; 8) smooth_muscle_cells; 9) T_cells (Figure 3(b)). Moreover, we evaluated the differential expression characteristics of the nine cell types (Figure 3(d)) and identified the expression of 7 SLC genes in the nine cells (Figures 3(e), 4(a)–4(o)). We found that the expression of 7 SLC genes was higher in hepatocytes and iPS_cells. This may be related to the involvement of SLCs in the conversion of CSCs to HCC [21].

### 3.4. Construction of a 7-SLC-Gene Signature Using the TCGA-LIHC Cohort

**Figure 5:**
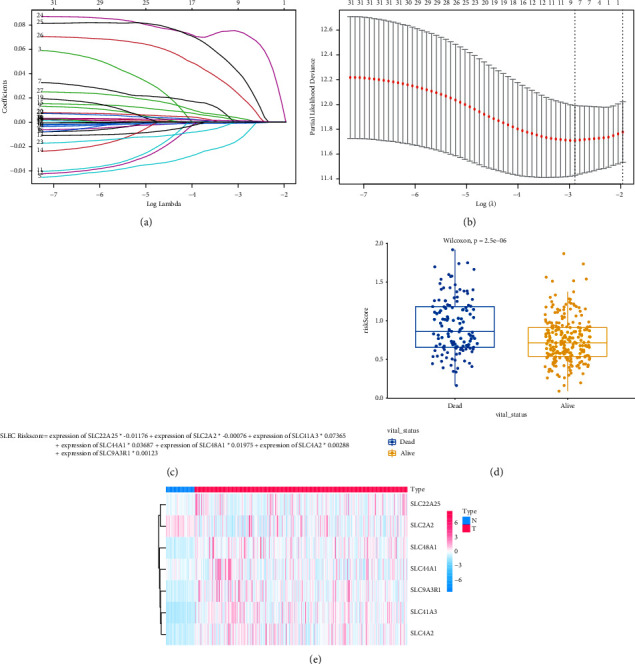
Building a seven-gene family signature for the TCGA cohort. (a) LASSO coefficients for genes associated with SLC. (b) LASSO Cox regression analysis with 1000 bootstrap replicates for selection of variables. (c) Establish a risk model related to the SLC gene. (d) Risk score in dead and alive samples. (e) Expression of 7 SLC genes in tumor tissues and normal tissues.

Due to the large sample size of the TCGA cohort, from the ICGC cohort, 231 samples were included in the validation set, and from the TCGA-LIHC cohort, 365 HCC samples have been contained in the training set. LASSO-Cox regression analysis was performed on 31 SLC genes related to prognosis (Figures 5(a) and 5(b)), and seven genes were finally selected for constructing an SLC-gene-based risk model. The risk scores of patients were evaluated using the following formula: SLC risk score = expression of SLC22A25^*∗*^−0.01176 + expression of SLC2A2^*∗*^−0.00076 + expression of SLC41A3 ^*∗*^0.07365 + expression of SLC44A1^*∗*^0.03687 + expression of SLC48A1^*∗*^0.01975 + expression of SLC4A2^*∗*^0.00288 + expression of SLC9A3R1^*∗*^0.00123 (Figure 5(c)).

**Figure 6:**
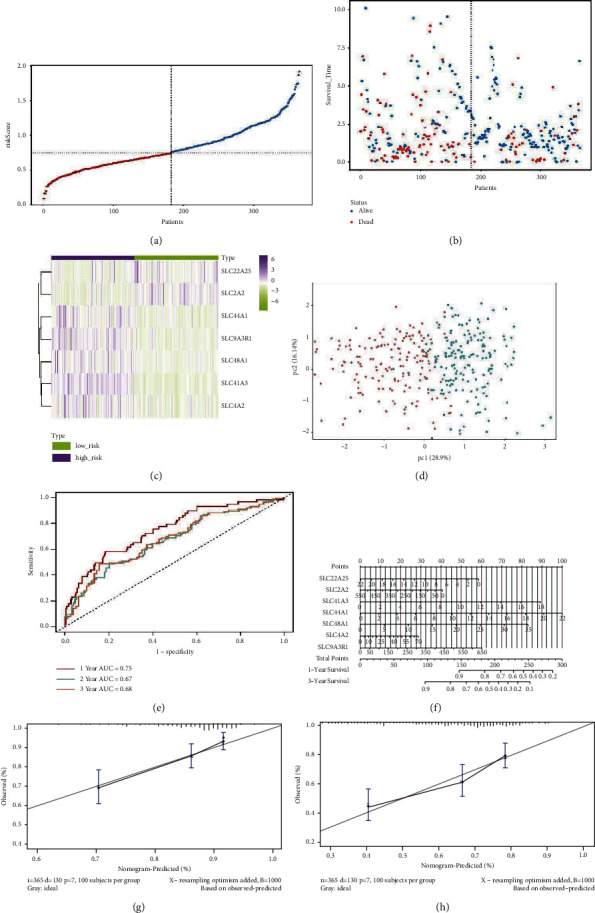
Prognostic analysis of the 7-gene signature model in the TCGA cohort. (a) The distribution of risk scores within the training cohort. (b) Vital status and follow-up time for patients in two risk groups. (c) A comparison of normal and tumor tissues expressing the SLC gene. (d) PCA plot of the training cohort. (e) ROC curve analysis of the seven-gene signature predicts overall survival in the training cohort. (f) The nomogram is based on the seven-gene signature. (g, h) Nomogram calibration curves for LIHC patients from TCGA cohorts predict an overall 1-and3-year survival.

The risk scores were significantly higher in patients who were deceased than in those who survived (*P* value <0.001) (Figure 5(d)). The heat map in Figure 5(e) shows a comparison of tumor and normal tissues in the expression levels of the 7 SLC-related genes. Patients in the training cohort were categorized into low- and high-risk groups by their median risk score (Figure 6(a)), and a higher risk score meant a greater likelihood of a shorter survival or death (Figures 6(b) and 6(c)) demonstrated seven SLC genes' expression profiles in the two risk groups. Principal component analysis showed a bidirectional distribution of patients in the different risk groups (Figure 6(d)). The predictive performance of the risk score was assessed based on time-dependent ROC curves, with AUC values of 0.75, 0.67, and 0.68 for 1, 2, and 3 years, respectively (Figure 6(e)), indicating that patients with HCC were accurately predicted to survive by the SLC-gene-based signature. A prognostic nomogram was additionally developed (Figure 6(f)), and calibration curves demonstrated that the prediction of 1-and3-year OS was similar to the ideal curve, indicating that patients with HCC were accurately predicted by the nomogram (Figures 6(g) and 6(h)).

### 3.5. Verification of the 7-Gene Signature in the ICGC Cohort

**Figure 7:**
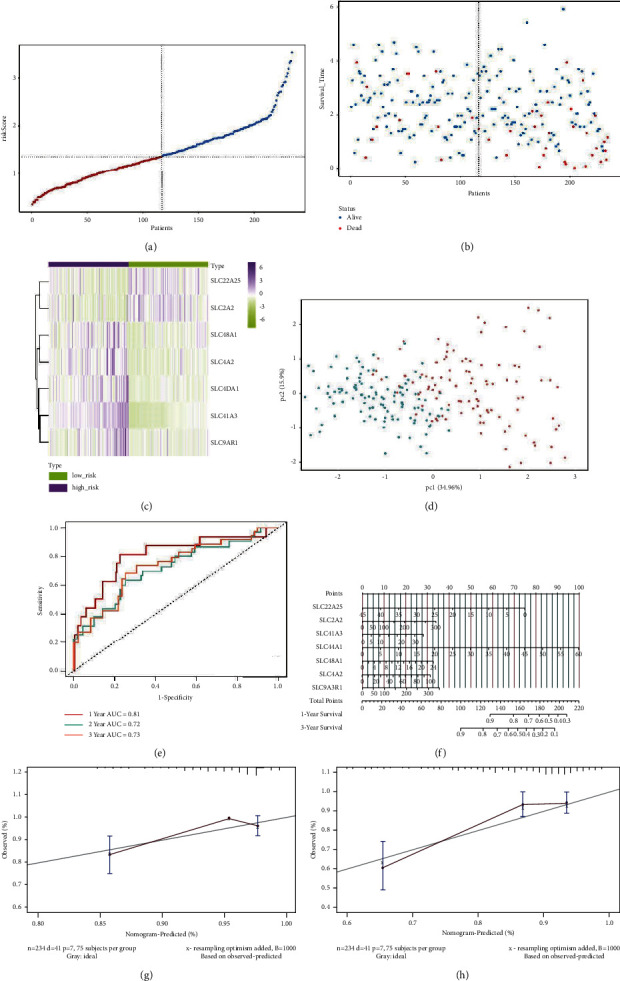
Validation of the 7-gene signature model in the ICGC cohort. (a) The distribution of risk scores within the testing cohort. (b) Vital status and follow-up time for patients in two risk groups. (c) A comparison of normal and tumor tissues expressing the SLC gene. (d) PCA plot of the training cohort. (e) ROC curve analysis of the seven-gene signature predicts overall survival in the training cohort. (f) The nomogram is based on the seven-gene signature. (g, h) Nomogram calibration curves for HCC patients from ICGC cohorts predict an overall 1- and 3-year survival.

**Figure 8:**
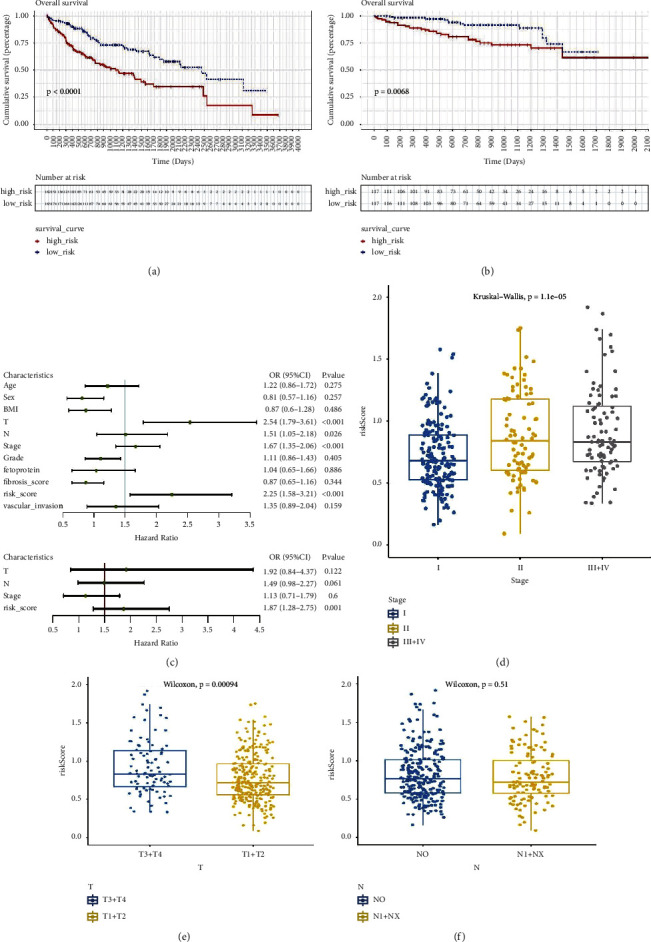
Correlation of risk models with clinical characteristics. In both TCGA (a) and ICGC (b) cohorts, Kaplan-Meier analysis revealed that high-SLC-risk HCC patients had a shorter overall survival. (c) Univariate survival analysis and multivariate survival analysis of clinical characteristics. (d–f) Risk score significantly correlated with stage (d), T category (e), but not with lymph node invasion (f).

The accuracy of the constructed risk signature was validated using data from the ICGC dataset though dividing patients into the groups at high- or low-risk as outlined above (Figure 7(a)). We found similar dot plots and heat maps to those in the TCGA cohort (Figures 7(b) and 7(c)). Using principal component analysis, patients in both subgroups showed a distinct distribution, similar to the TCGA cohort results (Figure 7(d)). As a result, the AUC of the 1-, 2-, and 3-year OS prediction were 0.81, 0.72, and 0.73, respectively (Figure 7(e)). Furthermore, a prognostic nomogram with calibration curves was constructed (Figures 7(f)–7(h)), and the nomogram accurately predicted the outcome of HCC patients. In addition, an analysis using Kaplan–Meier survival data (Figure 8(a), *P* < 0.0001) revealed that the high-risk group of the TCGA training cohort had a worse HCC prognosis for HCC. Validation results on the ICGC cohort were similar (Figure 8(b), *P*=0.0068).

### 3.6. Association of the Risk Signature with Clinical Characteristics

Considering the different clinical characteristics associated with prognosis in the two risk groups, we investigated the predictive ability of HCC-independent prognostic factors and the risk signature (Figure 8(c)). In addition, whether the clinical characteristics of HCC were associated with the risk signature was explored. TNM stage and tumor grade were both higher in the high-risk group (Figures 8(d)–8(f)). The current data indicated that the risk signature could be either used in combination with the clinical indicators available at present or serve as an independent prognostic factor.

### 3.7. GSEA for the Seven-Gene Signature

The GSEA technique was applied to the high-risk and low-risk groups of the TCGA training set for studying the functional enrichment of SLC genes.

**Figure 9:**
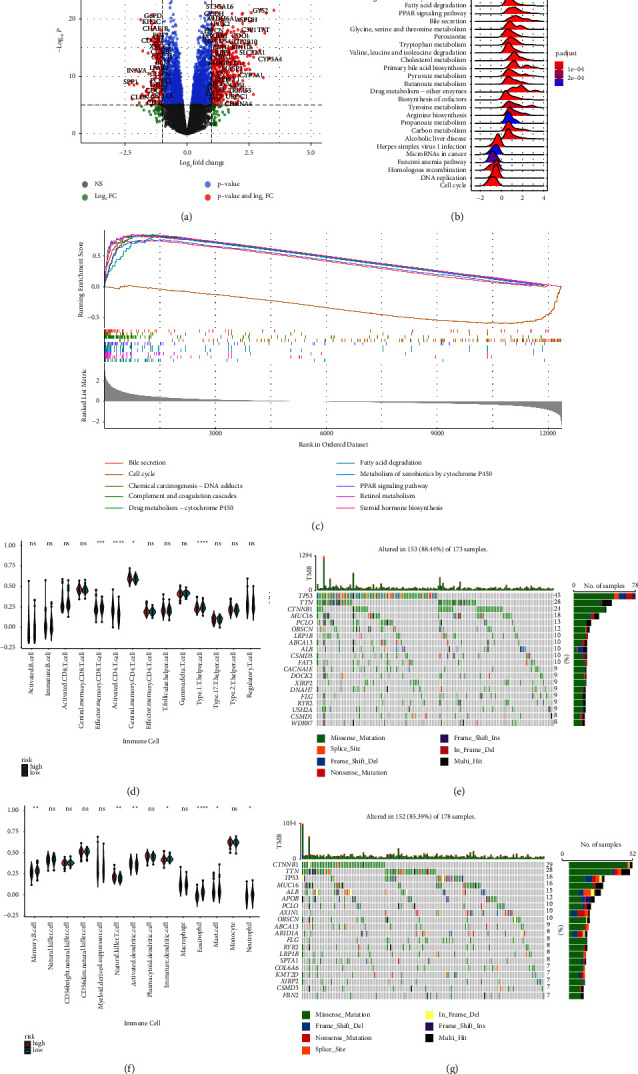
Functional analysis in the TCGA cohort. (a) The volcano plot of differentially expressed genes in low- and high- SLC risk groups based on TCGA-LIHC data. (b, c) GSEA of KEGG pathways in high- and low-risk of SLC groups. (d, f) The violin plots display the scores of 28 immune cells. (e, g) The top 20 mutational genes for high-(e) and low-risk (g) SLC groups of the TCGA-LIHC were shown.

**Figure 10:**
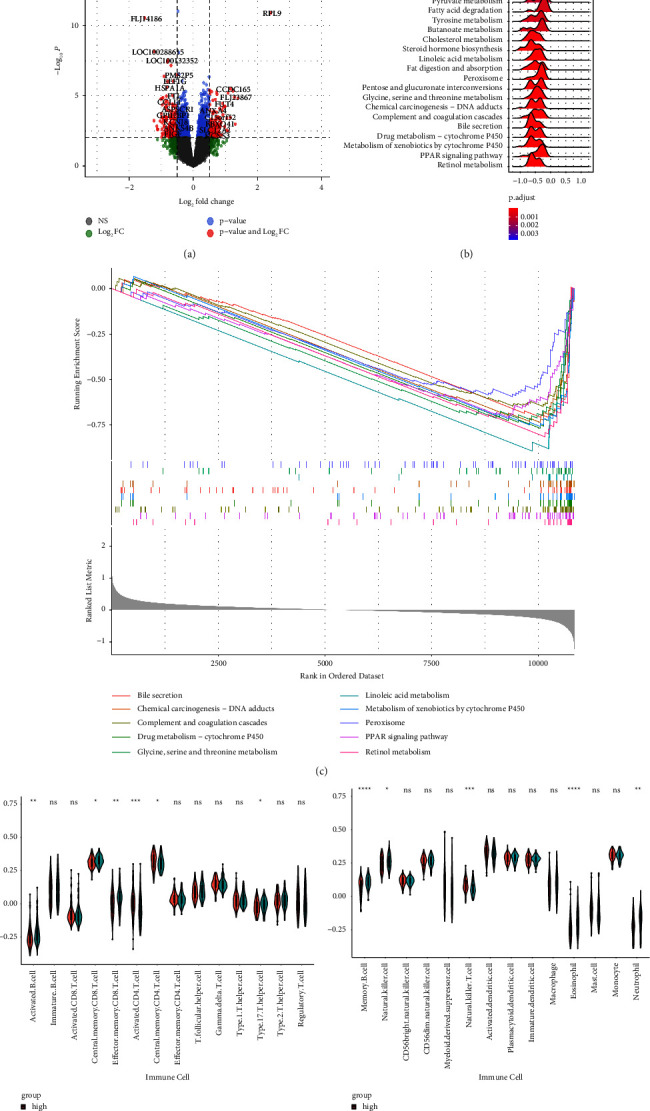
Functional analysis in the ICGC cohort. (a) Volcano plot of differentially expressed genes in low- and high-SLC risk groups based on data from ICGC. (b, c) GSEA of KEGG pathways in high- and low-SLC risk groups. (d, e) The violin plots display the scores of 28 immune cells in two risk groups.

The R package “limma” detected 12,363 differentially expressed genes (DEGs) in two risk groups (Figure 9(a)). The KEGG pathways were identified by GSEA, and complement and coagulation cascades, retinol metabolism, chemical carcinogenesis, and cytochrome P450 pathways were found to be significantly enriched (Figures 9(b) and 9(c)). On verifying these results in the ICGC cohort, 13,071 DEGs were identified (Figure 10(a)), and bile secretion, chemical carcinogenesis, complement and coagulation cascades, and cytochrome P450 pathways were significantly enriched (Figures 10(b) and 10(c)).

### 3.8. Correlation between Somatic Variations and the SLC Gene Signature

On waterfall plots, in the low- (Figure 9(g)) and high-SLC-risk (Figure 9(e)) subgroups, we identified the top 20 genes with the highest mutation frequencies. There were several genes frequently mutated in both groups, including TP53, TTN, CTNNB1, MUC16, PCLO, OBSCN, LRP1B, ABCA13, ALB, CSMD3, XIRP2, FLG, and RYR2. Among these genes, ten mutated genes (TP53, MUC16, PCLO, OBSCN LRP1B, ABCA13, CSMD3, XIRP2, FLG, and RYR2) showed a higher mutation frequencies and richer mutation profiles in the high-risk group. Nevertheless, two genes (CTNNB1 and ALB) with mutation had lower mutation frequency and narrower mutation profiles in the high-risk group.

### 3.9. The Relation of SLC-Gene-Based Signature to the Tumor Immune Microenvironment and Immune Cell Infiltration

**Figure 11:**
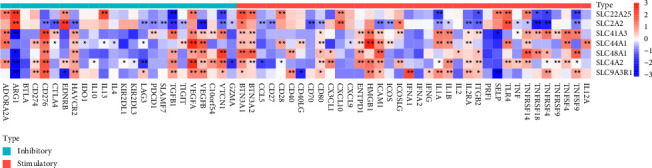
Correlations between SLC-related gene signature and immune checkpoint genes are mapped out as a heatmap. Red represents positive correlation, and blue represents negative correlation; the darker the color, the better the correlation.

The ESTIMATE algorithm was used to calculate the proportion of 28 infiltrating immune cells in different risk groups to examine the association between the SLC-gene-based risk signature and the immune microenvironment. In ICGC and TCGA cohorts, the proportion of activated CD4 T cells, central memory CD4 T cells, regulatory T cells, myeloid-derived suppressor cells, natural killer T cells, and activated dendritic cells were higher in the high-risk group, and that of effector memory CD8 T cells, activated B cells, memory B cells, natural killer cells, eosinophils, and neutrophils was lower in the high-risk group than in the low-risk group (Figures 9(d), 9(f) and 10(d), 10(e)). In addition, the relationship between SLC-related genes and immune checkpoint genes in patients with HCC was examined using TCGA pan-cancer data (Figure 11).

### 3.10. Prediction of Chemotherapy Treatment Response in Different Risk Groups

**Figure 12:**
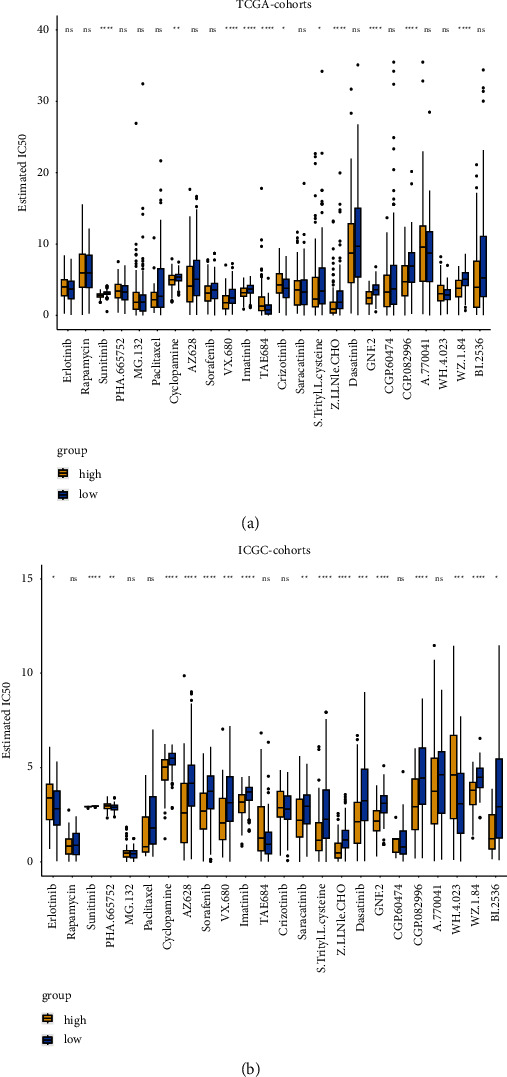
Analysis of cell line data from the GDSC database to evaluate IC50 for chemotherapeutics in high-risk and low-risk groups. (a) Treatment responses are evaluated by the new 7-SLC-gene signature prognosis score in TCGA cohort. (b) Treatment responses are evaluated by the new 7-SLC-gene signature prognosis score in ICGC cohort.

For patients with advanced liver cancer, chemotherapy is a standard treatment. We analyzed the effects of 24 chemotherapeutic agents on HCC in the GDSC database based on the drug “pRRophetic” software package to predict the IC50 of chemotherapeutic agents in HCC patients from different SLC risk score groups in the training and testing cohorts. Lower IC50 indicated higher sensitivity to chemotherapeutic drugs. In both TCGA and ICGC cohorts, a higher sensitivity of the high-risk group to sunitinib, cyclopamine, VX-680, imatinib, S-trityl-L-cysteine, Z-LLNIe-CHO, GNF-2, and CGP-082996 was observed, and WZ-1-84 than low-risk group (*P* < 0.05, Figures 12(a) and 12(b)).

### 3.11. Multidimensional Validation of the Key Genes in the HPA Database

**Figure 13:**
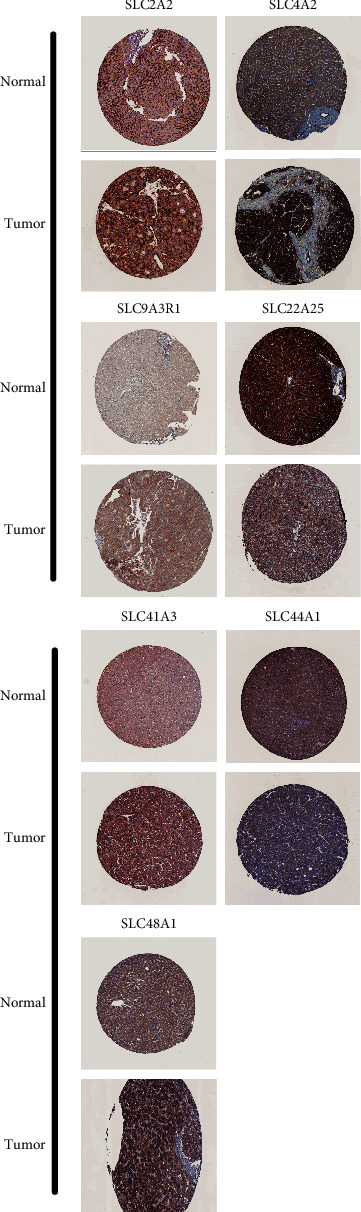
Protein expressions of SLC22A25, SLC2A2, SLC41A3, SLC44A1, SLC48A1, SLC4A2 and SLC9A3R1 in HCC specimens from the human protein atlas database.

To determine the protein expression of the 7 SLC genes, using the HPA database, images of IHC were analyzed. We found that SLC22A25 and SLC2A2 were intensely stained in normal tissues, whereas SLC44A1, SLC9A3R1, SLC48A1, SLC41A3, and SLC4A2 were deeply stained in HCC tissues (Figure 13). These results suggested that these seven genes were specific markers for SLC.

## 4. Discussion

HCC, a polygenic disease, is a complex, multistep process, and the late diagnosis of HCC is a major cause of poor prognosis. Developing new tools for diagnosing HCC can improve its prognosis. High-throughput sequencing facilitates precise treatment, and its use to mine genetic features to predict the prognosis of HCC has become a focus of research. According to a previous study, Zhang et al. constructed a 10-immune-related-lncRNA model using the TCGA and GSE76427 datasets [22]. Li et al. established a 6-gene model related to energy and amino acid metabolism through analyzing TCGA, GSE76427, and ICGC datasets [23]. Liao et al. applied the TCGA database and constructed a 4-gene model based on methylation-related differentially expressed lncRNAs (MDEs) [24]. Wu et al. analyzed TCGA data, 37 HCC tissues from patients in the Shandong Provincial Hospital, and 11 healthy liver tissues collected from surgically treated patients with liver trauma, and they developed a 4-gene model based on autophagy-related lncRNAs [25]. Song and Chu used the GSE16757, GSE14520, and ICGC datasets and built a 4-gene model based on autophagy-related lncRNAs [26]. Jiang et al. developed a hypoxia-related10-gene model using the TCGA, GSE14520, and ICGC datasets [27]. Despite relatively limited studies of SLC proteins in recent decades, the SLC superfamily is now known to be involved in tumourigenesis, including apoptosis, invasion, proliferation, metastasis, chemoresistance, and other cancer-related processes. Overexpression or suppression of SLC may offer novel strategies for diagnosis, treatment, or prognosis [28]. Two TS-SLC genes, SLC29A1 (ENT1) and SLC8A1 (NCX1), are downregulated in tumor cells (TCS) via the EMT-induced zinc finger E box binding homology box 2 (ZEB2)/transforming growth factor (TGF)-BR/nuclear factor (NF)-kB pathway, or miR-223 in HCC, respectively [29]. As a result of enhanced amino acid uptake by SLC38A1 and SLC7A5 (LAT1), and in HCC and TCS grows faster due to YAP/TAZ pathway activation [30]. The association between metal ion-mediated tumorigenesis and regulation of various metal transport proteins, including DMT1 (SLC11A2) for iron transport in HCC has been found [31]. The SLC13A5 gene encodes NaCT, which is seen as a sodium-coupled citrate transporter. NaCT plays a role in fatty acid synthesis, cellular glycolysis, gluconeogenesis cholesterol synthesis, and mitochondrial energy production in the liver [32]. A previous study observed that in liver samples from patients with obesity with insulin resistance and NAFLD, the mRNA expression of SLC13A5 was significantly increased, and was correlated with hepatic steatosis [33]. At the inner mitochondrial membrane, the SLC25A13 gene encodes aspartate-glutamate carrier 2 (AGC2) to facilitate the calcium-dependent exchange of cytoplasmic glutamate with mitochondrial aspartate. The SLC25A13 mutation could not be compensated by other transporter systems in the liver, which would also lead to HCC [34]. In HCC and SLC1A5 directly regulates the mTOR pathway, subsequent growth of HCC cells, and survival signals [35]. Thus, these studies suggested that SLC plays a role in the development and progression of HCC. In this study, the SLC family genes were comprehensively analyzed in HCC and SLC genes associated with the clinical features of HCC were identified via WGCNA. In addition, a 7-gene prognostic model of SLC (SLC22A25, SLC2A2, SLC41A3, SLC44A1, SLC48A1, SLC4A2, and SLC9A3R1; univariate Cox and LASSO regression algorithms) was designed and validated. The overall survival of training and validation cohort patients was consistently lower in the high-risk group, suggesting that the SLC-based signature assessment of HCC prognosis was accurate and generalizable. Furthermore, ROC analysis was performed to validate the sensitivity and specificity of the prognostic signature.SLC2A2 encodes glucose transporter protein 2 (GLUT2), which is associated with glycolysis and gluconeogenesis in the liver via the HNF4a-GLUT2 pathway that can affect the uptake and utilization of glucose by HCC cells and is involved in the systemic metabolism of cancer cachexia [36, 37]. The SLC4A2 gene encodes bicarbonate-chloride anion exchange protein 2 (AE2), which mediates proton leakage across the Golgi membrane and allows the Golgi apparatus to act as a proton reservoir in cancer cells, thereby regulating the pH microenvironment of TCS and promoting tumourigenesis and progression [38]. Malfunction of the acid-base homeostasis caused by SLC4A2 can also affect mitochondrial gradients and trigger ROS damage, leading to apoptosis, proliferation, and morphological alterations [39]. SLC9A3R1 encodes the sodium-hydrogen exchange regulator protein (NHERF1) and directly interacts with the PTEN pathway, and its deletion results in increased cell proliferation and Akt activation. Therefore, NHERF1 plays a tumor-suppressive role [40]. NHERF1 regulates Wnt signaling through maintaining a low level of *β*-catenin protein activation [41]. SLC9A3R1 regulates cancer cell proliferation and metastasis by enhancing PTEN levels to stimulate autophagy, subsequently inhibiting the PI3K-AKT1-MTOR pathway [42]. The SLC22 family proteins are known as “drug” transporters. This family of organic ion transporters mediated the excretion of drugs, endogenous substances, and environmental toxins in vivo, including the subgroups of OATs, OCTs, and OCTNs. The OATS4 member SLC22A25 is associated only with bound hormones, making it a relatively single specific transporter protein [43]. SLC22A25 is found in the liver, wherein high co-localization of glucuronide and sulfate is found with androgens and other gonadal steroids [44]. SLC41A3 encodes a mitochondrial Na^+^-dependent Mg^2+^ efflux system that regulates the intracellular Mg^2+^ homeostasis [45]. Mg^2+^ binds to various proteins and is involved in various cellular functions, including genome stabilization and immune responses [46]. Aberrant Mg^2+^ levels in cancers have been detected and this could promote cancer progression [47]. In several GEO (GSE36376, GSE22058, GSE64041, GSE76427, GSE63898, GSE14520, GSE54236) and ICGC (ICGC-LIRI) datasets, an increase in SLC41A3 level was found in tumor tissues compared to healthy tissues. A study by Liu et al. demonstrated that HCC patients with low levels of SLC41A3 expression have significantly better outcomes (OS). Compared to healthy tissues, LIHC had a significantly lower DNA methylation level of SLC41A3, which may account mainly for a high-expressed SLC41A3 in tumor tissues [48]. SLC44A1 encodes choline transporter-like protein 1 (CTL1) and is found in both plasma and mitochondrial membranes. SLC44A1 transports choline in a sodium-ion-non-dependentmoderate-affinity manner [49]. CTL1-mediated choline transport is a critical step in synthesizing phospholipids that form a plasma membrane. Apoptosis can be induced by inhibiting choline uptake [50]. Cancer cells have enhanced choline uptake via CTL1, which promotes membrane phospholipid synthesis and cell proliferation. Therefore, CTL1 could be a new target molecule for cancer therapy [51]. SLC48A1 is a heme transporter mainly found in endosomes and is involved in the transport of heme iron during iron metabolism. It encodes a facilitator transporter protein (HRG-1), which regulates the V-ATPase activity, enhances glucose transporter-1 (GLUT-1) transport, increases glucose uptake and lactate production, and promotes insulin-like growth factor I receptor (IGF-1R) transport [52]. Furthermore, overexpression of SLC48A1 promotes invasion, migration, and glycolysis, and cancer cell growth, which are associated with less favorable outcomes [53]. Still, this study had certain limitations. We utilized a public database to conduct a retrospective bioinformatics analysis, but it would be more convincing if the SLC-gene-based risk signature was cross-validated in more samples. In addition, the specific biological role of the seven prognostic SLC genes in HCC should be validated via molecular and animal experiments.

## 5. Conclusion

In conclusion, the 7-gene signature based on SLC genes showed a satisfactory accuracy and generalizability in predicting the survival outcomes of patients with HCC. In addition, in the tumor microenvironment, the signature was related to the tumor immune status and infiltration of different immune cells. Therefore, this study provided novel insights into developing SLC-based treatment strategies for HCC.

## Data Availability

All data generated or analyzed during this study are included in this article.TCGA (https://portal.gdc.cancer.gov/repository) ICGC databases (https://dcc.icgc.org/projects/LIRI-JP) and GEO database for GSE149614 dataset (https://www.ncbi.nlm.nih.gov/geo/query/acc.cgi?acc=GSE149614).
